# Proximity- or Conformation-Induced Caspase Activation?

**DOI:** 10.1371/journal.pbio.0030198

**Published:** 2005-05-10

In 1972, John Kerr, Andrew Wyllie, and Alistair Currie introduced the word “apoptosis” (from the Greek for apples falling off trees) to describe a special form of cell death. Apoptosis, or programmed cell death, is a normal physiological process in which specific signals trigger cells to self-destruct in a carefully choreographed manner. Without apoptosis, we would have paddles for hands, rather than individual fingers. And without apoptosis, we would be at much greater risk of developing cancer, since apoptotic mechanisms destroy cells that have taken the first steps toward tumor formation.

Cells apoptose when the positive signals needed for their survival are withdrawn or when negative signals tell the cell to commit suicide. A cell undergoing apoptosis shrinks and develops “blebs”—small bubbles—on its surface, its mitochondria break down, and its genomic DNA breaks into fragments. Finally, the dead cell is engulfed by phagocytic cells, thus avoiding any inflammation in surrounding tissues.

Apoptosis can be triggered by external and internal signals, but in both the extrinsic and intrinsic pathway of apoptosis, once a signal has been received, the next molecular step is caspase activation. These proteases, which cleave proteins at specific sites, fall into two classes. Initiator caspases activate themselves before proteolytically activating the effector (or executioner) caspases, which degrade numerous cellular proteins, thus causing cell death. All the caspases are made as inactive enzymes (zymogens); they are much too dangerous to be stored as active enzymes.[Fig pbio-0030198-g001]

**Figure pbio-0030198-g001:**
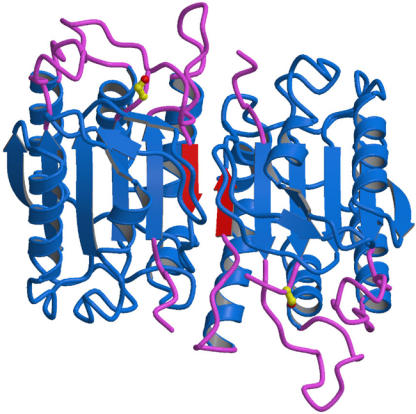
Experiments with artificially joined molecules of caspase 9—which normally exists as monomers (above)—suggest that caspase 9 can't activate cell death pathways by dimerization alone

Yigong Shi and colleagues are studying the mysterious process of initiator caspase activation—the molecular mechanism of effector caspase activation is fairly well understood. The autocatalytic activation of caspase-9, an initiator caspase in the intrinsic pathway of apoptosis, is mediated by the assembly of the apoptosome, a heptameric complex containing Apaf-1 (apoptotic protease activating factor 1) and cytochrome c. Caspase-9 activation is currently explained by the induced proximity model in which the apoptosome, by increasing the local concentration of caspase-9, promotes its homodimerization (the formation of a complex containing two caspase-9 molecules) and subsequent autoactivation.

To test this model, Shi and coworkers engineered caspase-9 so that it exists in the cell all the time as a homodimer—wild-type caspase-9 usually exists as monomer—and then examined its catalytic activity. They found that although the engineered, dimeric caspase-9 was more active in in vitro assays and induced more cell death when expressed in cells than the wild-type enzyme, its activity was only a fraction of that of Apaf-1-activated wild-type caspase-9. Furthermore, its activity was not stimulated by Apaf-1, unlike that of the wild-type enzyme. Importantly, they also show that the crystal structure of their engineered caspase-9 closely resembled that of wild-type caspase-9, indicating that the changes they made did not cause any significant changes in the protein.

Overall, their results led the researchers to suggest that the dimerization of caspase-9 may be qualitatively different from the Apaf-1-mediated activation of caspase-9, and that dimerization may not be the major mechanism behind the activation of caspase-9. Instead, they suggest that a shape (conformational) change is induced in caspase-9 when it binds to the apoptosome and that this change drives its activation. This “induced conformation” model provides an alternative model to the induced proximity model for initiator caspase activation, but, as the researchers note, the two models need not be mutually exclusive. Indeed, only the determination of high-resolution structure of the apoptosome will unravel exactly how caspase-9 and other initiator caspases are activated.

